# Knighthoods for Hospital Secretaries

**Published:** 1916-11-11

**Authors:** 


### THE EDITOR'S LETTER-BOX.

### Knighthoods for Hospital Secretaries.

*To the Editor of* The Hospital.

Sir,—A calling, it is argued in your article of last week, must be proved worthy before its representatives may be distinguished by titular honours. Thus, that of an actor, of the manager of some large stores, or of the director of a railway, has reached a point of excellence calling for recognition in this way, but not so that of the chief executive officer of a hospital.

But hospital secretaries, although they should possess many of the qualities that make successful actors, stores managers, and railway directors, do not worry themselves much about decorations; with their splendid opportunities of log-rolling it is rather to their credit than otherwise that they have hitherto escaped. But now and then, at a time when a man who has done good service is nearing the end of his working days, some recognition would seem suitable, even if the recipient has risen from the ranks.

But the most important part of the article deals with the desirability of improving the class from which hospital secretaries are recruited. Merely to raise the rates of remuneration would not keep out unsuitable men; an educational test such as that imposed by the Civil Service is of doubtful value as a means to secure the right people. Under what system can the level of hospital men be raised?

To be a man of breeding and education is not sufficient; the voluntary system makes it necessary that in addition to other qualities the secretary must have something of the showman and the shopkeeper in his composition. In short, personality is such an important factor that it is difficult to blame a committee for selecting the man most likely to be successful in the majority of his many different functions.

The subject is an important and interesting one, and the present is not a bad time to discuss it fully, especially as a number of the lower posts on the administrative side are filled by people who can claim little consideration because of length of service.

You may be able to extend your remarks and make suggestions as to general rules as to appointments on the administrative side; perhaps also hospital folk themselves may have something to say on the subject.—I am, yours faithfully, A London Secretary.

